# Investigation on the Distribution Characteristics of Chinese Continuing Education Based on the Community Detection Algorithm in Complex Networks

**DOI:** 10.1155/2022/8149395

**Published:** 2022-08-27

**Authors:** Yuping Lai, Qin Yuan, Qinming Yu

**Affiliations:** ^1^School of Foreign Language and Trade, JiangXi University of Engineering, Xinyu, Jiangxi 338000, China; ^2^School of Big Data and Computer, JiangXi University of Engineering, Xinyu, Jiangxi 338000, China; ^3^College of Humanities and Management, Heilongjiang University of Traditional Chinese Medicine, Heilongjiang 150040, China

## Abstract

In order to closely fit the characteristics of continuing education, the development of continuing education teaching activities under the network background should not only be combined with the characteristics of professional adult education but also make reasonable use of modern teaching models in the actual teaching process. Based on the community detection algorithm in complex networks, this article makes thorough research and analysis on the complexity of Chinese continuing education by using complex network technology. By establishing the characteristics of vertex degree distribution, average path length, and clustering coefficient of complex networks, it is confirmed that Chinese continuing education has scale-free network characteristics and small-world network characteristics. The three aspects of relationship strength comprehensively analyze the information dissemination speed, scope, interpretation, and application; through the combination of the ant colony algorithm and complex network technology, multiple information dissemination paths are abstracted in Chinese continuing education. The research shows that the application of complex network algorithms can effectively improve the speed and quality of continuing education in China. It is found that the government should increase the number of adult education projects and improve the level of project categories, form key adult education research basis to promote the diversification of research subjects, expand the space for adult education projects to balance regional and provincial differences and attach importance to basic research on adult education, and integrate applied research.

## 1. Introduction

**Figure 1:**
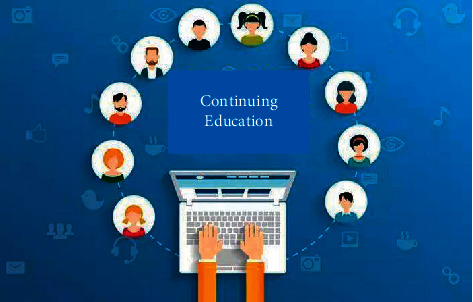
The structure of continuing education.

Since the 1990s, the development and utilization of the Internet and large-scale database systems have greatly reduced the cost of information dissemination and network resource development. It creates more material and spiritual wealth. With the development of information technology and its promotion and application in the field of continuing education, major changes have taken place in the content, form, method, and organization of continuing education, as shown in Figure 1.

Because of its huge role in talent training, continuing education accounts for a large proportion of higher education. Today, with the continuous updating of information technology and network technology, the cause of education reform is relying on information construction and network application to carry out plans. There are steps to change, and it also puts forward more requirements for the reform and development of continuing education. The network education model is very different from the traditional education model. It is an advanced product combining education and network technology, which not only inherits the advantages of traditional education, but also keeps pace with the times. The development of continuing education and teaching activities under the network background should not only be combined with the characteristics of professional continuing education, but also make reasonable use of modern teaching models in the actual teaching process, closely fit the characteristics and requirements of continuing education, and improve continuing education. The quality and optimization of the teaching effect play a favorable role [1, 2]. Kiss analyzed the resource status of the education and training vocational education network and identified the areas where the network was deficient [3]. Diana illustrated the merits of theorizing transition as nonlinear, continuous, dynamic, fluid, and ongoing in order to better understand the interplay of context on the lived experiences of indigenous and older adult students [4]. As an interdisciplinary subject formed by mathematics, physics, computer, and biology, network science has unparalleled advantages in using network methods to study scientific problems, and it has been given more and more attention by scholars at home and abroad [5–8].

Continuing education can provide opportunities for learners who leave school and enter society to continue to receive education, consolidate professional knowledge and improve work ability, and meet the needs of learners to learn knowledge and skills. King et al. noted that one of the ways health professionals maintain and acquire the necessary knowledge and skills is through continuous professional development [9]. Subsequently, many scholars have further studied the relationship between continuous learning and professional development [10–14]. The pace of development in modern society is getting faster and faster. People's time is compressed, again and again, resulting in more and more fragmented time. Traditional continuing education models such as TVU and correspondence cannot keep up with the needs of learners, and networked continuing education models can break the routine, overcome the problems of time and space, and enable learners to better plan and use time and resources to continue their education. Networked continuing education avoids the one-to-one teaching mode in traditional continuing education, and learners and teachers are no longer unable to conduct character attacks due to limited time. The “timer” control is used to achieve character attacks. Whether the attack ends depends on whether the character's “life value” is zero. This series of questions, thinking, and conclusions guides students to gradually complete the project. Exploring the evolution law of the network in this complex network world and obtaining the hidden and valuable potential information resources has attracted the extensive attention of scholars. Related results can be found in [10–15].

From the perspective of technical expression, continuing education in the information age presents the characteristics of digitization, networking, intelligence, multimedia, personalization, and virtualization. (1) The carrier of knowledge dissemination presents digital characteristics. The traditional use of paper, blackboard, video, or audio recording as the means of knowledge dissemination is transformed into digital information processed and transmitted by a computer, and the media such as text, symbols, graphics, sound, and images are digitized. This is what happens after continuing education uses information technology. Fundamental change. In today's information age, the acquisition, transmission, and processing of knowledge have all developed in a digital direction. The practice has proved that digitally processed knowledge information has the characteristics of high fidelity, large storage capacity, fast dissemination speed, and wide audience. (2) The learning environment presents network characteristics. In the information age, continuing education and science and technology have developed simultaneously. The combination of computer technology and communication technology has brought human beings into a new network environment. People's learning has become more and more dependent on terminal devices such as computers, mobile phones, and laptops. Information technology has spread all over the world. Many computer systems with independent processing capabilities are connected through telecommunication lines and corresponding equipment to realize the sharing of educational resources. The networked exchange of digital knowledge and information has become more and more convenient and efficient, and e-mail, remote login, exchange forums, and remote defense have also penetrated into people's lives and studies. (3) Management and services are intelligent. Compared with traditional face-to-face education or off-the-job learning, continuing education in the information age makes more use of modern information technology and overcomes the drawbacks (high labor costs, cumbersome work, duplicate work, low efficiency, etc.), replaced by an intelligent information management system. The basic information of the objects participating in continuing education (educational education, job responsibilities, skill level, career planning, etc.), continuing education files, learning management and services, associate learning courses, learning status, learning reminders, notifications, etc. is organically combined. Through learning exchanges and other content, continuing education participants not only gain knowledge and skills, but also enjoy a full range of educational and learning services. The use of information technology in continuing education reflects the intelligent integration of learning and management [16–18]. Qazi et al. compared online learning among students in Brunei and Pakistan during COVID-19 [19]. Yoldoshev et al. discussed the process of using modern technology in language teaching in Uzbekistan and put forward some technical suggestions for the further development of foreign language teaching [20].

For a long time, actual networks such as communication networks, power networks, biological networks, social networks, and economic networks have been the research objects of different disciplines such as communication science, electric power science, life science, social science, and economic science, respectively [21–23]. In addition, there are also some branches of abstract network research, such as the network composed of abstract points and lines in the study of graph theory in mathematics, and the study of game theory in the use of related parties in games between multiple individuals or teams under specific conditions, strategies and implementing the corresponding strategy [24–27].

As an interdisciplinary subject formed by mathematics, physics, computer, and biology, network science has unparalleled advantages in using network methods to study scientific problems, and it has been given more and more attention by scholars at home and abroad. First, network science studies the commonalities between complex networks that appear to be different from each other and the general methods for dealing with them. The source of research problems in network science is various actual networks, and the common concepts, methods, and theories generated by them that can in turn provide macro guidance and specific means for the analysis and design of various actual networks. Second, network science attempts to build a bridge of communication between various existing research-related disciplines, so that the research on one network may also serve as a reference for the research on another network. In addition, many complex network problems cannot be effectively solved by a single discipline and require multidisciplinary collaborative efforts, and network science is such a multidisciplinary cross-platform. Network science studies the commonalities between complex networks that appear to be different from each other and the general methods for dealing with them. With the rapid development and wide application of Internet technology, artificial intelligence technology, and complexity science, complex network theory has received high attention in many research fields.

## 2. Community Detection Algorithm in Complex Networks

In the research of this article, the network research work is carried out in two steps. The first step is to establish different group networks based on the group system according to this research. The network uses different network community discovery algorithms and a proposed network community discovery method based on penetration theory to perform network clustering in turn and compare the results of network clustering; overall, the work of this article can be divided into network construction, network statistics, network clustering, and classification accuracy analysis. These two steps will use different network construction or splitting skills. A complex network not only has a high degree of complexity, but also has strong interdisciplinary characteristics, which is an emerging interdisciplinary subject.

### 2.1. Construction of the Network

According to the data collection and processing, the distribution of each group on the 500*∗*500 spatial grid can be obtained. In order to study the connections between each group, this article will construct the group network according to the following steps:

1. Each group in the group is taken as a node in the network, and the number of grids occupied by each group is used as the size of the node.
2. The distribution of any two groups in the group in the grid is considered: if their distributions do not intersect, then there is no connection between the nodes corresponding to the two groups; if there is an intersection, they are calculated at 500. The number of grids distributed in the *∗*500 grid is used as the weight of the connecting edge between the two nodes.
3. According to the definition of network nodes and edges in the above two steps, the network can be constructed.

### 2.2. Network Statistics

In network science theory, the out-degree of node *i* is the number of directed edges from this point, and the in-degree of node *i* is the number of edges that point to node *i* from other nodes. In this study, the studied network is an undirected network, so the degree here is defined as the number of edges connected to node *i*. Intuitively, the greater the degree of a node, the more “important” the node is in a sense. But in the eigenvector centrality that reflects the measure of node importance, the basic idea is that the importance of a node depends not only on the number (degree) of neighbor nodes, but also on the importance of neighbor nodes. If *A* is the eigenvector centrality of the node, then we have

(1) $$\begin{array}{c} x_{i}=c\underset{N⟶\infty }{\lim}\sum_{j=1}^{N}a_{ij}x_{j}, \end{array}$$

where *c* is the proportionality constant and *A* is the network adjacency matrix.

Then,

(2) $$\begin{array}{c} x={\left[x_{1},x_{2},\ldots x_{N}\right]}^{T}. \end{array}$$

Then, the above formula can be written as

(3) $$\begin{array}{c} x=cAx. \end{array}$$

The above formula means that *x* is the eigenvector corresponding to the matrix *A* and the eigenvalue, so it is called the eigenvector centrality. Therefore, the eigenvector centrality is the index that can best reflect the importance of nodes in the network. The larger the value, the more important the node is. In addition, modularity is a standard commonly used in recent years to measure the quality of community division. The network after grouping is compared with the corresponding null model to measure the quality of the grouping. Modularity is defined as follows:

(4) $$\begin{array}{c} Q=\underset{n⟶\infty }{\lim}\sum_{i=1}^{n}\left[\frac{e_{i}}{m}-{\left(\frac{k_{c_{i}}}{2m}\right)}^{2}\right], \end{array}$$

where *m* is the number of edges in the network, *e*_*i*_ is the number of edges in the community *i*, and *k*_*c*_ is the sum of the degrees of all points in the community *i*. The source of research problems in network science is from various actual networks, and the common conceptual methods and theories produced by it can in turn provide macro-guidance and specific means for the analysis and design of various actual networks in multidisciplinary cross-platforms.

### 2.3. Network Clustering

A random walk is defined as moving from one vertex to the next, selecting a neighbor of the current vertex as the next vertex with an equal probability. The basic idea is that communities are relatively dense subgraphs, so it is easy to “fall into” a community when performing random walks in the graph. The specific process is as follows: first, the adjacency matrix *A* corresponding to the network *G* is normalized by row, and then, the probability transition matrix *P* is obtained. Expressing this normalization process using matrix computation, it can be written as

(5) $$\begin{array}{c} P=D^{-1}A. \end{array}$$

In the formula, *A* is the adjacency matrix; *D* is the degree matrix.

Using the Markov property of the *P* matrix, it can be known that its elements to the power of *t* represent the probability of a random walk particle from node *i* to *j* through *t* steps. Second, the distance between two points *ij* is defined as follows:

(6) $$\begin{array}{c} r_{ij}=\left\|D^{-0.5}P_{i}^{t}-D^{-0.5}P_{j}^{t}\right\|,\\ \left\|D^{-0.5}P_{i}^{t}-D^{-0.5}P_{j}^{t}\right\|=\sqrt{\underset{n⟶\infty }{\lim}\sum_{k=1}^{n}\frac{{\left(P_{ik}^{t}-P_{jk}^{t}\right)}^{2}}{d\left(k\right)}}. \end{array}$$

The distance from community *C* to point *j*:

(7) $$\begin{array}{c} P_{Cj}^{t}=\frac{1}{\left|C\right|}\sum_{i\in C}P_{tj}^{t}. \end{array}$$

Then, the distance between communities is defined:

(8) $$\begin{array}{c} r_{C_{1}C_{2}}=\left\|D^{-0.5}P_{C_{1}}^{t}-D^{-0.5}P_{C_{2}}^{t}\right\|,\\ \left\|D^{-0.5}P_{C_{1}}^{t}-D^{-0.5}P_{C_{2}}^{t}\right\|=\sqrt{\underset{n⟶\infty }{\lim}\sum_{k=1}^{n}\frac{{\left(P_{C_{1}k}^{t}-P_{C_{2}k}^{t}\right)}^{2}}{d\left(k\right)}}. \end{array}$$

The specific steps of the algorithm are as follows:

The first step: each point is regarded as a community, and the distance between adjacent points (communities) is calculated.

**Step 1 .**

The two communities that minimize the following formula are selected and merged into one community:

(9) $$\begin{array}{c} \Delta \sigma \left(C_{1},C_{2}\right)=\frac{1}{n}\frac{\left|C_{1}\right|\left|C_{2}\right|}{\left|C_{1}\right|+\left|C_{2}\right|}r_{C_{1}C_{2}}^{2}. \end{array}$$

Initially, each vertex is assigned to its own community, and then the modularity *M* of the entire network is calculated; the first step: each community pair is required to be linked by at least one single edge. If two communities are fused together, the algorithm calculates the modularity changes caused by this.

**Step 2 .**

The group pair with the largest growth in *M* is taken and then fused. Then, a new modularity, *M*, is calculated for this cluster and recorded.

Steps 1 and 2 are repeated, each time fusing community pairs so that the maximum gain of *M* is finally obtained, and then the new clustering patterns and their corresponding modularity scores *M* are recorded. When all the vertices have been grouped into one giant cluster, it can stop. The algorithm then examines the records in the process and finds the clustering pattern that returns the highest *M* value.

Community partitioning based on principal eigenvectors is an algorithm that finds closely connected subnetworks in a network by computing the principal nonnegative eigenvalues of the network modularity matrix. The core of this method is the definition of the modularity matrix *B*:

(10) $$\begin{array}{c} B=A-P. \end{array}$$

In the formula, *A* is the adjacency matrix; *P*_*ij*_ is the probability of a connection between node *i* and node *j* in another network with the same number of nodes and the same degree of each node, but the connection of edges is random. Network science attempts to build a bridge of communication between various existing research-related disciplines, so that the research on one network may also serve as a reference for the research on another network. In the information technology period, the practical significance and practical application value of complex network research have gradually developed into a very challenging subject in scientific inquiry in the information technology period.

## 3. Investigation on the Distribution Characteristics of Continuing Education in China

For a long time, actual networks such as communication networks, power networks, biological networks, social networks, and economic networks have been the research objects of different disciplines, including communication science, electric power science, life science, social science, and economic science.

### 3.1. Analysis of the Number of Projects

**Figure 2:**
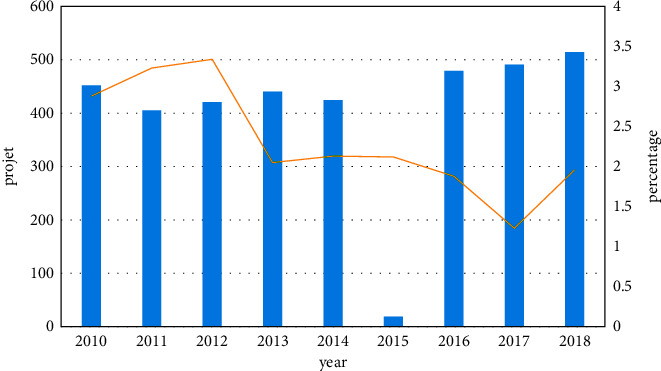
Project and percentage.

As shown in Figure 2, from 2010 to 2018, there were 4038 national educational science planning projects, including 92 adult education projects, accounting for 2.28%. Judging from the annual number of projects approved for adult education topics, although the number of projects approved in the past 9 years has fluctuated, it has generally remained stable. From 2010 to 2012, the number of projects approved for adult education showed a slight upward trend, reaching 14 projects in 2012, the highest value in the past nine years. In the following 6 years, the number of projects has stabilized at around 9. Among them, the number of adult education projects in 2017 was the least. In general, the number of adult education projects is relatively small, which is not conducive to the development of adult education scientific research.

The project approval rate is the percentage of the number of adult education projects approved in the total number of national educational science planning projects in that year. From 2010 to 2012, with the gradual increase in the number of adult education projects, the project approval rate also showed an increasing trend and reached its highest peak in 2012. Since 2012, except for 2017, the project approval rate fell to the lowest. The project approval rate in the remaining years did not change much, and the project approval rate fluctuated. The average project approval rate for adult education subjects was low. Only the project approval rate from 2010 to 2013 was higher than the average project approval rate, and the project approval rate in the past 6 years was lower than its average project approval rate. In addition, the average project approval rate of each discipline in the 14 subject groups (with the national defense and military education disciplines listed separately) funded by the National Education Science Plan is 1/14. It can be seen that the project approval rate of adult education projects is much lower than the average project approval rate of the 14 subject groups. This shows that adult education projects have not received enough attention, and social attention needs to be improved. In addition, many complex network problems cannot be solved effectively by a single discipline and require multi-disciplinary collaborative efforts, and network science is such a multidisciplinary cross-platform.

### 3.2. Category Analysis of Project-Approved Topics

The projects approved by the National Education Science Planning Office are divided into two levels: one is the national-level projects approved by the National Office of Philosophy and Social Sciences, which are divided into national major projects, national key projects, national general projects, and national youth fund projects; the other is representative. The Ministry of Education projects approved by the Ministry of Education include the key topics of the Ministry of Education and the Ministry of Education's youth special projects. The Ministry of Education's planning project was no longer established in 2004, so there are no data on such projects.

**Figure 3:**
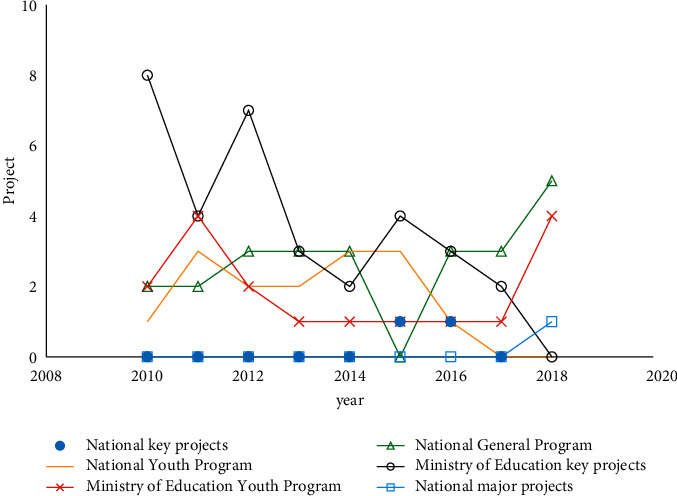
Comparison of project in each type.

**Figure 4:**
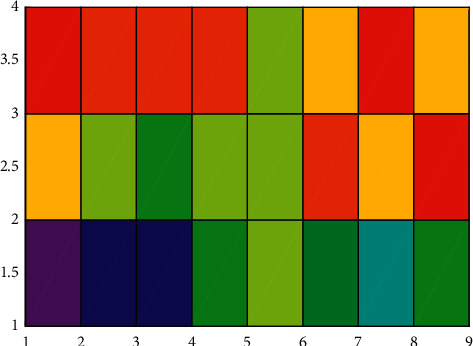
Data variation.

**Figure 5:**
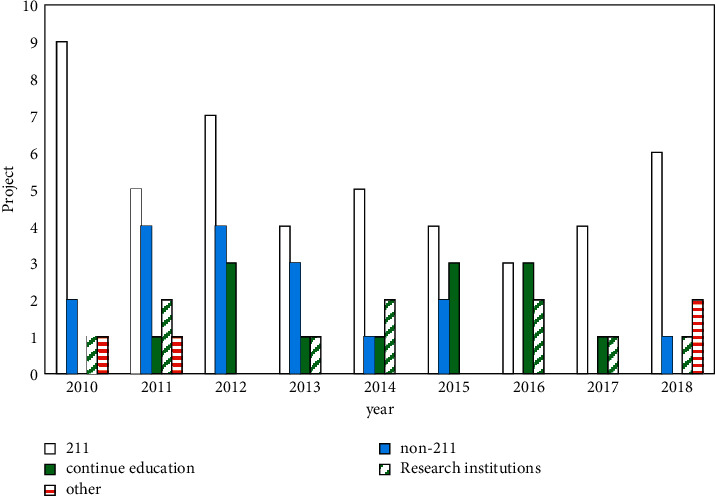
Project in different organizations.

**Figure 6:**
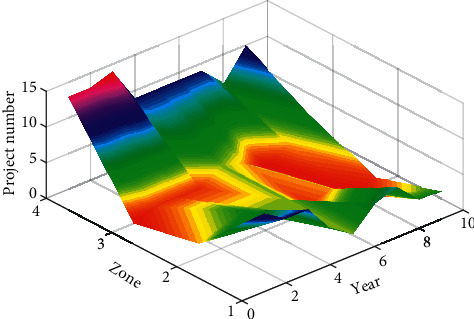
Project number.

**Figure 7:**
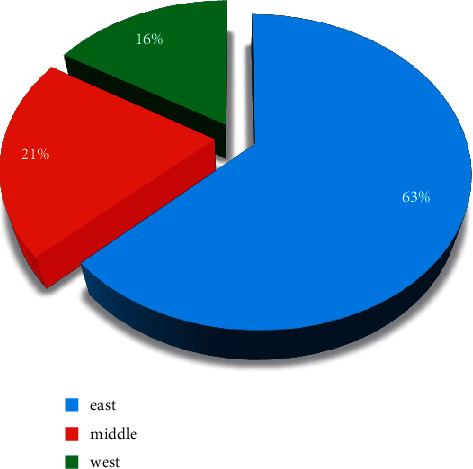
Percentages.

**Figure 8:**
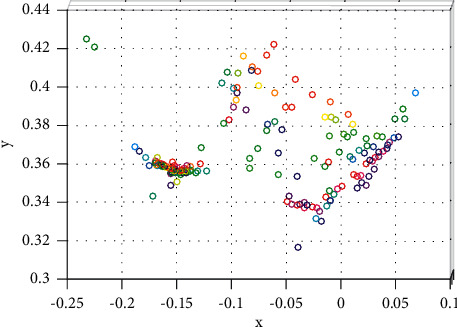
*x* and *y* variation.

In this article, the distribution characteristics of Chinese continuing education are presented by using the community algorithm. The experiments are actually relative to the presented method. The basic data, such as Figures 3to 8, are calculated based on community detection. To a certain extent, data such as the category distribution ratio and the proportion of the same type of adult education projects can reflect the basic situation of adult education project research. As shown in Figure 3, the total number of adult education projects from 2010 to 2018 is large, including major national projects, national key projects, national general projects, national youth fund projects, and key projects of the Ministry of Education. Judging from the category distribution ratio, the category distribution ratio of adult education subjects is similar to the overall category distribution ratio of national educational science planning projects. Among them, there are many adult education projects at the ministerial level. This shows that the development of adult education research is largely dependent on the administrative and financial support of the Ministry of Education. At the same time, among the adult education projects, there are national youth projects and youth projects of the Ministry of Education. This is conducive to promoting the development of the academic ability of young researchers and also reflects the country's emphasis on cultivating young adults in the main body of adult education research. Among the adult education subjects, the number of national major subjects and national key subjects is relatively small. In the past 9 years, there are only little subjects. Among them, East China Normal University has undertaken national major subject and national key subject. It is not difficult to see its role in adult education research as a core position in the field. There is also a national key project being undertaken by the education administrative department. The data variation is shown in Figure 4.

From the data in the last column of Figure 3, “Percentage of the same category,” it can be seen that in the past 9 years, adult education projects have included major national projects. Because major national topics are often forward-looking, comprehensive, and strategic around major theoretical and practical issues in national education reform and development, this can reflect that the country has recognized that adult education has the ability to serve the country's major needs, but regrettably, the number of national major subjects in adult education is still very small. Except for major national topics, other categories of topics account for less than the same category. This reflects to a certain extent that the research strength of adult education still has a lot of room for improvement compared with other educational disciplines, and it also reflects that adult education is in a disadvantageous position in the education discipline group.

### 3.3. Distribution of the Affiliated System of the Responsible Units of the Project

Taking into account the actual situation of adult education research, this article divides the affiliation system of the subject responsible unit into higher education institutions (which can be further subdivided into original “211” universities and nonoriginal “211” universities), scientific research institutions, educational administrative departments, adult schools (including the Open University, Radio and Television University, and advanced education schools), and 6 other (including vocational colleges and middle schools) categories. The corresponding statistical results are shown in Figure 5.

Judging from the proportion of the affiliation systems of various responsible units, colleges and universities are the main forces carrying out adult education research. In the past 9 years, they have undertaken 64 projects, accounting for 69.57% of the total adult education projects. This is largely due to the strong research strength and rich research resources of universities. Among the colleges and universities, the original “211” colleges and universities obtained a total of 17 projects, accounting for 18.48% of the total number of adult education projects. Judging from the annual change trend, the number of subjects awarded by the original “211” colleges and universities has shown a downward trend, from 4 items in 2012 to 1 item in 2018. The non-“211” colleges and universities with a large proportion of colleges and universities have obtained a total of 47 projects, accounting for 51.09% of the total number of adult education projects. It can be seen that non-“211” colleges and universities are very enthusiastic in the application of adult education projects, and have become the mainstay of adult education research, playing an increasingly important role. Adult schools have undertaken 13 projects in the past 9 years, accounting for 14.13%. The number of projects approved by scientific research institutions is second only to that of adult schools. In the past 9 years, a total of 10 projects have been awarded, accounting for 10.87%. Middle schools, higher vocational colleges, and educational administrative departments also undertake the project establishment, but the participation rate is low. It can be seen that the main body of adult education research in my country is becoming more and more diversified, and a research community is gradually being formed, which is dominated by colleges and universities, supplemented by adult schools and scientific research institutions.

### 3.4. Analysis of the Area Where the Responsible Unit of the Project Is Located

In order to understand the research level of adult education in various regions, Figure 6 counts the frequency of the regions where the units of adult education projects are responsible and the proportion to the total number of adult education projects. The number of projects approved in the eastern region far exceeds that in the central and western regions combined, and the number of projects in the eastern region is nearly four times that in the western region, which shows that the eastern region is the center of adult education and scientific research. The percentages between east, middle, and west are compared in Figure 7. In addition, in the past 9 years, the number and proportion of projects in the central region have shown a downward trend each year, and the level of adult education and scientific research in the eastern and central regions has been increasing. It should be noted that this article presents a community algorithm to investigate the distribution characteristics of Chinese continuing education. By using the proposed method, the corresponding results can be calculated, including a comparison of the project in each type, data variation, projects in different organizations, etc.

**Figure 9:**
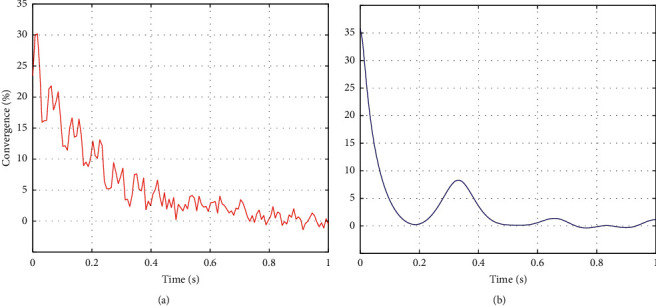
Convergence comparison: (a) conventional method and (b) proposed method.

Besides, through the frequency statistics of the provinces, autonomous regions, and municipalities to which 92 projects have been approved, it is found that most of the top-ranked provinces and cities in terms of the number of projects are in the eastern provinces and cities, and the two provinces and cities with the largest number of projects (Shanghai, 18, Zhejiang 16) all belong to the east. Hunan Province in the central region (7 projects) ranks third in the country in terms of the number of projects approved. This is largely due to the fact that Hunan Province has more specialized adult education research institutions and adult colleges and attaches great importance to adult education research. Except for Hunan Province, the number of projects approved in the rest of the central provinces is 3 or less. Only Shaanxi Province (4 items), Chongqing Municipality (3 items), and Sichuan Province (3 items) ranked high in the western region. Among the provinces and regions without adult education projects, the provinces and regions in the western region accounted for more than half. All these prove that there is a large gap in scientific research level between the eastern, central, and western regions of my country. Similar to the regional distribution, there are also differences in the distribution of provinces, regions, and cities. In the past 9 years, 22 provinces, autonomous regions, and municipalities have approved projects, and the remaining 9 provinces, such as Liaoning, have no projects. Although 22 provinces, autonomous regions, and municipalities participated in the project establishment, they were mainly distributed in Shanghai, Zhejiang, Hunan, Jiangsu, and Shandong. They had undertaken 53 projects in total, with more than half of the total number of projects approved. The rapid development of adult education, the high level of economic development, and the fact that there are more colleges and research institutions with strong scientific research strengths are not unrelated. Shanghai has the most projects approved, with a total of 18 projects, accounting for 19.57%. The total number is more than the total number of projects approved in the next seven provinces and municipalities, including Tianjin, Jilin, and Gansu. It plays a leading role in adult education research. Even in Zhejiang and Hunan, which are among the top three, there is a difference of nine projects between the two. There is a difference of 17 in the number of projects approved in Shanghai and Fujian Province, which are located in the eastern region. All in all, the regional distribution of adult education projects in my country is characterized by “concentration and unevenness,” and there is a huge disparity in the number of projects approved in different regions, and even different provinces and cities in the same region, which should be highly regarded. The *x* and *y* variation is shown in Figure 8. Besides, the convergence is compared in Figure 9, where both the conventional method and the proposed method are plotted. As can be seen, the convergence of the proposed method is much better than that of the conventional method.

## 4. Conclusion

Many systems in real life can be abstracted into network models, and researchers can understand the structure and functions of real systems through the study and analysis of network models. Based on the community detection algorithm in complex networks, this article makes thorough research and analysis of the complexity of Chinese continuing education by using complex network technology. By establishing the characteristics of vertex degree distribution, average path length, and clustering coefficient of complex networks, it is confirmed that Chinese continuing education has scale-free network characteristics and small-world network characteristics. The three aspects of relationship strength comprehensively analyze the information dissemination speed, scope, interpretation, and application; through the combination of the ant colony algorithm and complex network technology, multiple information dissemination paths are abstracted in Chinese continuing education. Research shows that the application of complex network algorithms can effectively improve the speed and quality of continuing education in China. In addition, the article also conducts a quantitative analysis of the number of adult education projects in the National Education Science Planning from 2010 to 2018, the types of projects, the responsible units for the projects, and the research topics, in order to sort out the status and characteristics of adult education research in the past nine years. Based on quantitative statistics, relevant conclusions are drawn and the following suggestions are put forward: increase the number of adult education projects and improve the category level of the projects; form a key research base for adult education to promote the diversification of research subjects; expand the space for adult education projects to balance regional and provincial differences; emphasis on basic research on adult education and integration of applied research; based on domestic adult education research, with an international perspective.

## Data Availability

The data used to support the findings of this study are available from the corresponding author upon request.

## References

[1] Darville R., Milana M., Desjardins R. (2018). Book review: global networks, local actions: rethinking adult education policy in the 21st century. *Political Economy of Adult Learning Systems: Comparative Study of Strategies, Policies and Constraints*.

[2] Tian L., Tang W. (2020). A probe into the reform of the talent training mode of “Internet adult higher education”. *International Journal of Social Sciences in Universities*.

[3] Kiss A. (2020). Collaboration and networking in adult education and training. A case study. *Acta Universitatis Sapientiae, Social Analysis*.

[4] Diana A. (2022). Indigenous and older adult higher education students: challenging systemic and linear transitions for inclusion. *International Journal of Educational Research Open*.

[5] Huo J., Yang X. (2020). The perfect combination of rural community adult education and modern network course resources construction. *International Journal of Educational Management*.

[6] Li C. (2021). Research strategies for sustainable development of adult higher education in medical colleges. *Advances in Vocational and Technical Education*.

[7] Liu Z. (2020). An analysis of educational administration teaching management model based on “Internet+” adult higher education. *International Journal of Educational Technology*.

[8] Guan S., Blair E. (2020). Correction to: Chinese adult higher education as a heterotopia. *Higher Education*.

[9] King R., Taylor B., Talpur A. (2021). Factors that optimise the impact of continuing professional development in nursing: a rapid evidence review. *Nurse Education Today*.

[10] Chen M. C., Lu S. Q., Liu Q. L. (2022). Uniqueness of weak solutions to a Keller-Segel-Navier-Stokes model with a logistic source. *Applications of Mathematics*.

[11] Yu Hsiu L. H. (2020). Learning Chinese as the united nations language? Implications for language learning motivation and identity in adult higher education. *International Journal of Higher Education*.

[12] Wang H., Yang X., Wang S., Li S., Guo Z. Integration of entrepreneurship innovation education into adult higher education.

[13] Wang H., Yang X., Wang S., Li S., Guo Z. Research on the implementation of innovation and entrepreneurship education in adult higher education.

[14] Rowland M. L. (2019). Book review: spirituality, community, and race Consciousness in adult higher education: Breaking the Cycle of racialization, by T. P. Westbrook. *Adult Education Quarterly*.

[15] Peterson S., Shepherd M., Farrell J., Rhon D. I (2022). The blind men, the elephant, and the continuing education course: why higher standards are needed in physical therapist professional development. *Journal of Orthopaedic & Sports Physical Therapy*.

[16] Suleymanova A. (2022). The main characteristics of stages of content and technology in continuing pedagogical education. *Open Journal of Social Sciences*.

[17] Wang X., Yu X., Guo L., Liu F., Xu L. (2020). Student performance prediction with short-term sequential campus behaviors. *Information*.

[18] Guo Q., Zhu Z., Lu Q., Zhang D., Wu W. (2020). A dynamic emotional session generation model based on Seq2Seq and a dictionary-based attention mechanism. *Applied Sciences*.

[19] Qazi A., Naseer K., Qazi J. (2020). Conventional to online education during COVID-19 pandemic: do develop and underdeveloped nations cope alike. *Children and Youth Services Review*.

[20] Yoldoshev O. A., Mamadayupova V. S., Matenov R. B., Toshmatov O. S. (2020). Journal of critical reviews impact of innovative modern technology in teaching languages in Uzbekistan. *Journal of Critical Reviews*.

[21] Wu E. Q., Zhou M. C., Hu D. (2022). Self-paced dynamic infinite mixture model for fatigue evaluation of pilots’ brains. *IEEE Transactions on Cybernetics, early access*.

[22] Pourdamghani N., Knight K. (2019). Neighbors helping the poor: improving low-resource machine translation using related languages. *Machine Translation*.

[23] Bote-Curiel L., Muñoz-Romero S., Gerrero-Curieses A., Rojo-Álvarez J. L. (2019). Deep learning and big data in healthcare: a double review for critical beginners. *Applied Sciences*.

[24] Chen M. C., Lu S. Q., Liu Q. L. (2021). Uniqueness of weak solutions to a Keller–Segel–Navier–Stokes system. *Applied Mathematics Letters*.

[25] Chen Y., Ma Y., Mao X., Li Q. (2019). Multi-task learning for abstractive and extractive summarization. *Data Science and Engineering*.

[26] Chen M. C., Lu S. Q., Liu Q. L. (2020). Uniform regularity for a keller–segel–Navier–Stokes system. *Applied Mathematics Letters*.

[27] Xiao X. (2018). Analysis on the employment psychological problems and adjustment of retired athletes in the process of career transformation. *Modern Vocational Education*.

